# Standardized, high-efficiency protocol for *in vitro* murine jawbone osteoclast isolation and differentiation

**DOI:** 10.3389/fimmu.2026.1971046

**Published:** 2026-10-29

**Authors:** Gulnaz Guliyeva, Marina Kleim, Mai A. M. Ibrahim, Antonio Maccataio, Ulrike Steffen, Georg Schett, Manuel Weber, Bettina Grötsch

**Affiliations:** 1 Department of Internal Medicine 3 – Rheumatology and Immunology, Friedrich-Alexander-Universität Erlangen-Nürnberg and Uniklinikum Erlangen, Erlangen, Germany; 2 Deutsches Zentrum für Immuntherapie (DZI), Friedrich-Alexander-Universität Erlangen-Nürnberg and Uniklinikum Erlangen, Erlangen, Germany; 3 Department of Oral and Cranio-Maxillofacial Surgery, Friedrich-Alexander-Universität Erlangen-Nürnberg and Uniklinikum Erlangen, Erlangen, Germany

**Keywords:** high-efficiency protocol, *in vitro* osteoclastogenesis, jaw bone, osteoclast, standardized

## Abstract

Osteoclasts derived from distinct skeletal sites exhibit site-specific differences in osteoclastogenic potential, reflecting variations in developmental origin, local microenvironment, and regulatory signaling pathways. Jawbones differ from long bones in their remodeling characteristics, reflecting their neural crest origin, intramembranous development, and functional integration with the periodontal ligament, which together shape distinct stromal programs, mechanotransduction cues, and bone turnover set points. These differences are especially relevant when studying diseases such as periodontitis, where alveolar bone resorption is a hallmark feature. However, due to the lack of standardized protocols for the isolation and culture of jawbone derived osteoclasts, most *in vitro* studies rely on bone marrow–derived osteoclasts, which incompletely recapitulate craniofacial osteoclast biology and may therefore limit translational relevance. Here, we present a comprehensive protocol for the generation of osteoclasts derived from murine jawbone tissue, followed by downstream *in vitro* processing and analytical evaluation. The protocol is complemented by standardized methods to assess osteoclast differentiation and functional resorptive capacity using calcium phosphate coated surfaces. By enabling the reliable generation and characterization of osteoclasts from craniofacial bone, this approach provides a physiologically relevant *in vitro* model for studying periodontitis and other oral bone pathologies, thereby enhancing the mechanistic understanding of site-specific osteoclast biology, and supporting the development of targeted therapeutic strategies.

## Graphical Abstract

**Figure:**
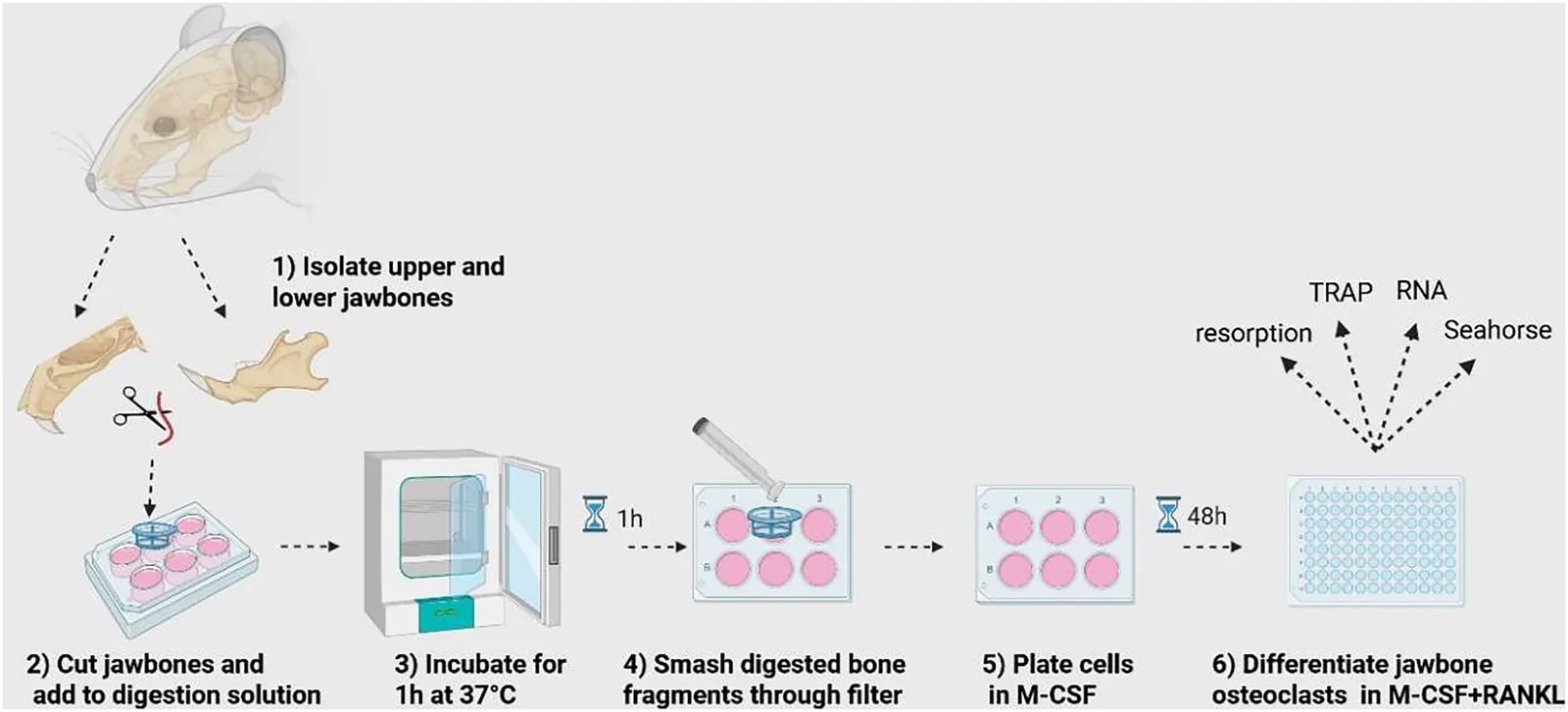
Schematic overview of the experimental workflow for jawbone isolation and osteoclast generation. Generated by Biorender.com

## 1 Introduction

Periodontitis is a highly prevalent, polymicrobial, dysbiosis-driven inflammatory disease and a leading cause of tooth loss worldwide (1). It is characterized by chronic immune activation and osteoclast-mediated destruction of alveolar bone. Disease progression is accompanied by a shift in the oral microbiome toward a pathogenic community enriched in microbial ligands such as β-glucans, mannans, and glycoproteins (2). These pathogen-associated molecular patterns (PAMPs) are recognized by pattern recognition receptors (PRRs) on host immune cells, linking microbial dysbiosis to inflammation and alveolar bone destruction (3).

Osteoclasts are multinucleated cells uniquely responsible for bone resorption and are essential for maintaining skeletal homeostasis through their coordinated activity with osteoblasts (4, 5). Disruption of this balance contributes to pathological bone loss in diseases such as osteoporosis, arthritis, and periodontitis (6). Osteoclast differentiation from monocyte-macrophage precursors is critically dependent on receptor activator of nuclear factor-κB ligand (RANKL) and macrophage colony-stimulating factor (M-CSF) (7, 8). Efficient osteoclast differentiation requires coordinated activation of receptor activator of nuclear factor-κB (RANK) and immunoreceptor tyrosine-based activation motif (ITAM) signaling pathways. Their synergistic activation maintains PLCγ2 phosphorylation, induces sustained intracellular Ca²^+^ oscillations, and drives NFATc1-dependent transcriptional programs essential for osteoclast differentiation (9, 10). Beyond canonical RANK signaling, osteoclasts directly sense microbial and inflammatory stimuli through PRRs. PRRs coupled to ITAM-bearing adaptor molecules further amplify osteoclastogenic signaling under inflammatory conditions, promoting differentiation and bone-resorbing activity (11, 12).

Accumulating evidence demonstrates that osteoclasts are not functionally uniform throughout the skeleton. Jawbone-derived osteoclasts originate from distinct precursor populations and display differences in differentiation, inflammatory responsiveness, and resorptive capacity compared with long bone-derived osteoclasts, reflecting site-specific developmental programs and local microenvironmental cues (13). While RANKL-induced NFATc1 activation represents the central osteoclastogenic pathway, microbial signals within the periodontal environment are increasingly recognized as important modulators of osteoclast precursor activation and inflammatory bone loss (14). However, how these microbial cues integrate with canonical osteoclastogenic signaling in jawbone-derived osteoclasts remains poorly understood, highlighting the need for physiologically relevant *in vitro* models (15).

*In vivo* models, including ligature- and bacteria-induced periodontitis, have established the critical role of microbial dysbiosis in driving osteoclast activation and alveolar bone loss (16). However, mechanistic studies remain limited by the lack of *in vitro* systems that preserve the unique characteristics of jawbone-derived osteoclasts. Most current approaches rely on long bone-derived precursors, which do not fully reflect the developmental origin, microenvironmental conditioning, or receptor repertoire of osteoclasts residing in the chronically microbe-exposed alveolar bone niche (17). Although such systems cannot recapitulate the full complexity of the periodontal environment, including stromal, immune, and microbial interactions, they provide an essential platform to investigate intrinsic jawbone-specific osteoclast responses and enable direct comparison with long bone-derived cells.

Although few protocols for the isolation of jawbone-derived osteoclast precursors have been described (18–20) detailed methodological information on their isolation and following *in vitro* differentiation is not consistently provided. This may limit the straightforward adoption and reproducibility of these approaches across laboratories and highlights the need for a more comprehensively described and standardized workflow for studying jawbone-specific osteoclast biology and host–microbe interactions.

Here, we establish a robust and reproducible protocol for the isolation, differentiation, and functional characterization of murine jawbone-derived osteoclasts. This method provides a physiologically relevant *in vitro* platform for studying jaw-specific osteoclast biology, inflammatory bone loss, and microbial sensing pathways. By bridging the gap between complex *in vivo* disease models and conventional long bone-based osteoclast cultures, this approach enables mechanistic investigation of site-specific osteoclast responses to inflammatory and microbial stimuli while supporting future therapeutic development and reducing reliance on animal experiments.

## 2 Materials and equipment

### 2.1 Jawbone isolation

- Donor mouse: common wildtype strain, e.g., C57BL/6, 6–10 weeks old
- Tweezers and Scissors (11,5 cm/9 cm 91100-12/14058-09)
- Scalpel (optional)
- Ethanol (75%)
- 1x PBS, pH 7.2 (without calcium and magnesium)

### 2.2 Tissue digestion and single cell suspension

• Osteoclast medium:

 Alpha MEM (Gibco #32571093).

 + 1% PenStrep (Gibco #15140122).

 +10% fetal calf serum (FCS; Bio&Sell #FBS.S.0615, Lot: BS.79605337.ST).

• Digestion solution:

 Osteoclast medium (37 °C).

 +1 mg/ml Collagenase type II (Sigma #C 2-22-1g).

 +1 mg/ml Dispase II (Sigma #D4693-1G).

- 100 µm flat cell strainer (e.g. Corning #CLS431752-50EA).
- 40 µm cell strainer (pluriStrainer-43-50040-50).
- 6-well Plate (Greiner bio-one 657160).
- 5ml syringes (BD 03626817).

 Note: Prepare digestion solution fresh immediately before use. Stir for up to 1h to dissolve the collagenase and dispase.

 Note: FCS is a highly sensitive and critical component for osteoclast differentiation. Therefore, we strongly recommend testing each batch before use to ensure consistent and reliable differentiation results.

### 2.3 *In vitro* cultivation

• Osteoclast medium (37 °C).

 + Recombinant murine soluble RANKL (Peprotech #315-11C).

 + Recombinant M-CSF (Peprotech #315-02-50).

• 6-well Plate (Greiner bio-one 657160).

• 96-well plate (Greiner bio-one 655940).

 Note: For standard osteoclast culture conditions used for TRAP staining and Seahorse experiments, we recommend using 10% FCS supplemented with 10 ng/ml RANKL. For CaP lysis assays, higher stimulation conditions are required, using 20% FCS and 20 ng/ml RANKL. M-CSF is maintained at 30 ng/ml across all experimental conditions.

### 2.4 Materials and reagents for supportive methods

#### 2.4.1 Tartrate-resistant acid phosphatase staining

- Acid Phosphatase (TRAP) Kit (Sigma-Aldrich, 387A-1KT).
- Acetone
- 37% Paraformaldehyde (PFA).
- 1x PBS, pH 7.2 (without calcium and magnesium).
- 1.5ml Eppendorf tube
- 15ml falcon
- Light microscope equipped with camera (Keyence, BZ-X710)

#### 2.4.2 Analyses of resorptive capacity on calcium phosphate coated plates

- 96-well plate (Corning, #3989)
- 0.12 M Na_2_HPO_4_
- 0.05 M Tris
- 0.20 M CaCl_2_ adjusted to pH 7.4
- 0,5% (w/v) silver nitrate
- Light microscope equipped with camera (Keyence, BZ-X710)

#### 2.4.3 Seahorse assay materials

- Seahorse XF Mito Stress Test Kits (Agilent Technologies, 103016-100)
- Seahorse XF Glycolysis Stress Test Kit (Agilent Technologies, 103020-100)
- Seahorse XF Calibrant Solution 500ml (Agilent Technologies, 100840-000)
- Seahorse XF DMEM assay medium, pH 7.4, 500ml (Agilent Technologies, 103575-100)
- Seahorse XFe96/XF Pro FluxPak (Agilent Technologies, 103792-100)

## 3 Methods

The objective of this study was to establish a simple, reproducible, and efficient method for the isolation, differentiation, and characterization of jawbone-derived osteoclasts for *in vitro* investigation of dental diseases, including periodontitis. This protocol provides a robust platform for studying osteoclast-specific signaling pathways and enables controlled activation and inhibition experiments under defined conditions.

The method involves the isolation of osteoclast precursor cells from murine jawbone tissue, followed by enrichment of monocyte/macrophage populations via adhesion-based selection and their subsequent differentiation into mature, functional osteoclasts.

### 3.1 Murine alveolar jawbone isolation

1. Euthanize the donor mouse (C57BL/6NRj, 6–10 weeks old) using CO_2_ inhalation, followed by cervical dislocation.

 Note: Use mice up to 10 weeks of age for osteoclast isolation, as older animals may result in reduced osteoclast differentiation efficiency and impaired cell fusion, leading to suboptimal culture quality.

 Note: Because osteoclast numbers obtained from jawbone tissue are limited, we recommend pooling tissue from four mice per experiment to obtain enough cells. To reduce inter-animal variability, keep cells from each mouse separate throughout the isolation procedure and combine only at the time of seeding, if required by the experimental design.

2. Sterilize the mouse fur on the head by spraying 70% ethanol onto the surgical area.

 a. Open the mouth and make an initial perioral skin incision to facilitate access. Carefully dissect the overlying soft tissue and proceed with bilateral cuts along the maxillary region until the upper jaw is fully exposed (**Figure 1**).

**Figure 1:**
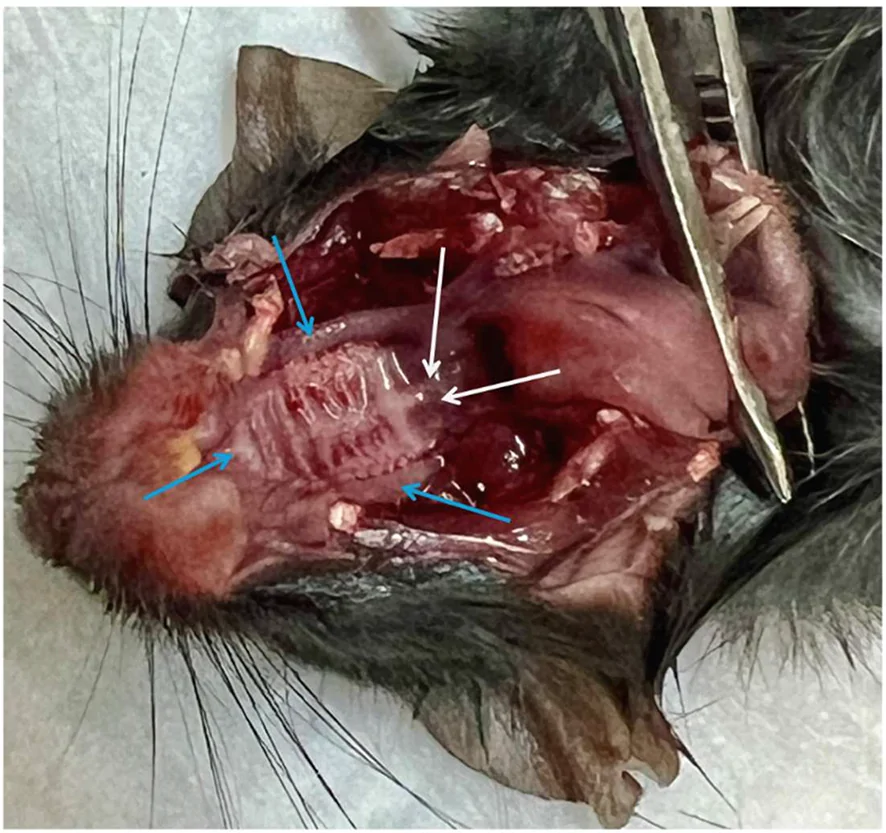
Stepwise isolation of the murine upper jawbone (maxilla). Blue arrows mark the initial incision sites used to loosen the maxilla from the surrounding tissues. White arrows indicate the final detachment step, in which a cut parallel to the palate is made to completely separate the maxilla from the remaining craniofacial structures.

 b. To isolate the upper jawbone, including the soft palate and teeth, use scissors to cut along all surrounding borders (**Figure 1**). Make additional cuts anteriorly near the incisors and posteriorly toward the condylar region. Perform a final incision parallel to the jaw and gently remove the intact upper jawbone (see video) (**Figure 2A**).

**Figure 2:**
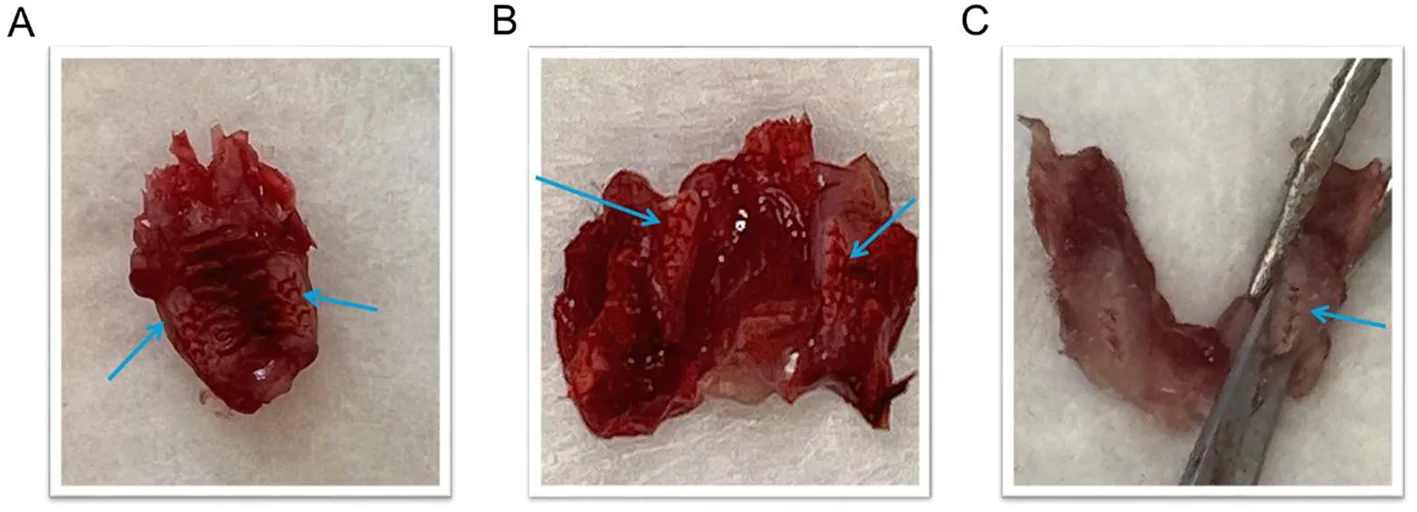
Representative isolated jaw specimens. **(A)** Maxilla and **(B)** mandible with attached soft tissue. The teeth (arrows) and palatal tissue of the maxilla were sectioned and removed prior to tissue digestion. **(C)** Illustration of the transection of the coronal portions of the teeth using scissors.

 Note: Handle the tissue with care throughout the dissection to avoid fracturing the delicate maxillary bone structure.

 c. Isolate the mandible and transect the coronal portions of the teeth using small, sharp scissors (**Figures 2B, C**) to expose the molar region. Carefully dissect and remove the overlying soft tissues with a scalpel under controlled conditions.

 d. Perform a longitudinal incision in the mandible parallel to the inferior border of the jaw to facilitate resection of the bony segment (see video).

 e. Carefully excise the residual soft tissues, including the lingual musculature, integumentary structures, masticatory musculature, and remaining dentition, to obtain a cleaned mandibular specimen with adherent gingival tissues.

### 3.2 Jawbone tissue digestions and single cell suspensions

*Note: All reagents and laboratory equipment used in contact with cells are required to be sterile, and handling has to be performed using appropriate aseptic techniques*.

1. Remove all residual soft tissue including palate. Using scissors, carefully cut off only the upper, exposed portion of the teeth, leaving the tooth roots embedded in the jawbone. This can be done without damaging or fracturing the jawbone. Briefly immerse the upper and lower jawbones in 70% ethanol for 30 seconds for decontamination, and transfer the bones to 1.5 ml Eppendorf tubes containing 500 µl sterile 1× PBS, with each tube containing the maxilla and mandible from a single mouse. Store the samples in 1× PBS until use in the tissue digestion procedure.
2. Prepare a 6-well plate by placing a 100 µm Corning cell strainer into each well, transfer the jawbones into the wells, and cut them into small fragments using sterile scissors prior to incubation (**Figure 3**).
3. Proceed by adding 5 ml of digestion solution per well, ensuring that the bones are fully submerged.
4. Incubate the bone fragments in digestion solution at 37 °C under 5% CO_2_ for 1 hour.
5. After 1 hour of incubation, remove the plate from the incubator. Gently dissociate the digested bone fragments by pressing them through the 100 µm cell strainer using the sterile rubber end of a syringe plunger.
6. Filter the resulting cell suspension through a 40 µm cell strainer into a 50 ml Falcon tube to remove any remaining undigested tissue and bone fragments.
7. Centrifuge the cell suspension at 1400 rpm for 5 minutes at room temperature.
8. Carefully discard the supernatant and resuspend the cell pellet in 5 ml of osteoclast culture medium supplemented with 5 ng/ml M-CSF.
9. Transfer the cell suspension to a fresh well of a 6-well plate, maintaining one well per mouse to ensure samples remain separated throughout the culture period. Incubate the plate at 37 °C in a humidified atmosphere with 5% CO_2_.

**Figure 3:**
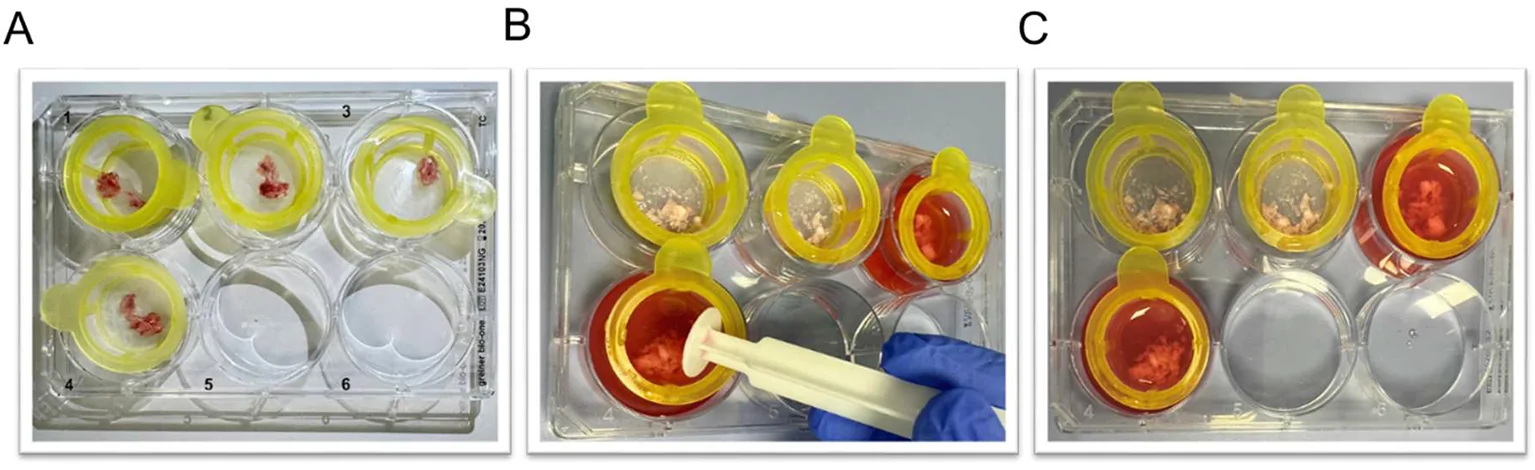
Jawbone digestion and homogenization workflow. Representative images of jawbone fragments at different stages of processing. **(A)** Before homogenization and prior to the addition of digestion solution. **(B)** After 1 hour of incubation in digestion solution, during homogenization. **(C)** Final homogenized suspension immediately before filtration to obtain a single-cell suspension. From the first two wells, medium was removed to better see the digested jawbone fragments.

Note: Inclusion of low-dose M-CSF during the first monocyte incubation step enhances cell survival and preserves differentiation potential, resulting in more robust osteoclast formation.

### 3.3 Protocol for the isolation and differentiation of long-bone osteoclasts used for comparison with isolated jawbone osteoclasts

1. Isolate the femurs and tibias from one mouse and carefully remove all residual soft tissue.

Briefly immerse the bones in 70% ethanol for 30 seconds for surface decontamination. Subsequently, rinse the bones thoroughly with sterile PBS.

2. Expose the bone marrow cavity by cutting off both ends of the femur and tibia. Place each bone into a 0.5 mL Eppendorf tube with a small hole in the bottom. Insert the 0.5 mL tube into a 1.5 mL Eppendorf tube containing 200 µL PBS.

3. Centrifuge for 10 seconds at 10.000 rpm to flush the bone marrow from the femur and tibia into the 1.5 mL tube containing PBS.

4. Resuspend the bone marrow cells thoroughly by gentle pipetting and transfer the cell suspension to the appropriate culture wells.

Culture and differentiate the bone marrow cells into osteoclasts under the same conditions described for jawbone-derived osteoclasts.

### 3.4 *In vitro* differentiation of jawbone osteoclasts

1. After 2 days of culture, collect the non-adherent cells from each well while maintaining the samples from individual mice separately. To do so, gently pipette the culture medium up and down to dislodge and suspend the non-adherent cells, then transfer the cell suspension through a 70 µm cell strainer into a 50 ml Falcon tube. Each well contains cells derived from the upper and lower jawbones of a single mouse (**Figure 4**).

**Figure 4:**
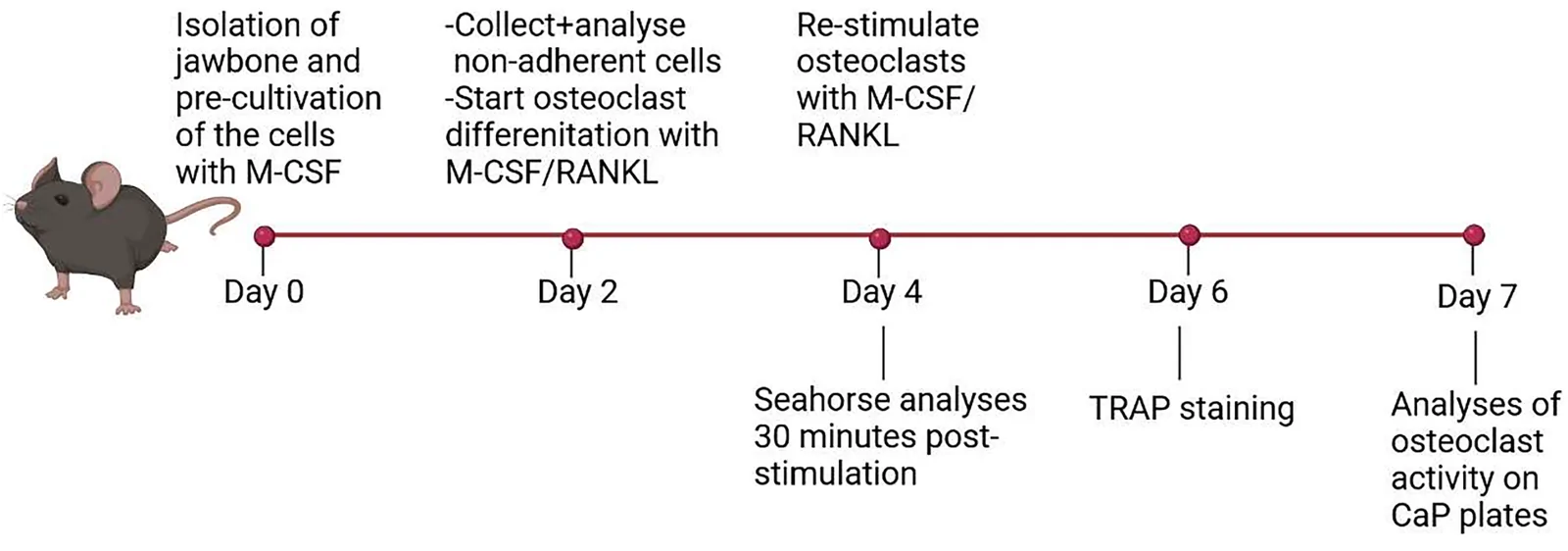
Time course of jawbone osteoclast differentiation. Generated with Biorender.com.

*Note: After filtration, cell suspensions from multiple mice may be pooled into a single 50 ml Falcon tube for downstream processing. Pooling cells from four mice per tube is recommended.*

2. Centrifuge the cells for 5 min at 1400 rpm at room temperature.

3. Centrifuge the collected cells and resuspend the cell pellet in 1 ml of osteoclast culture medium. Determine the number of viable cells using a Neubauer hemocytometer or an automated cell counter after preparing a 1:10 dilution.

*Note: A typical yield is approximately 3–5 x10^6^ viable cells per mouse.*

4. Seed the cells at a density of 2 × 10^5^ cells/ml in osteoclast culture medium. For 96-well plates, dispense 200 µl of cell suspension per well. Culture the cells in medium supplemented with 30 ng/ml recombinant murine M-CSF and 10 ng/ml recombinant murine soluble RANKL to promote osteoclast differentiation (**Figure 4**).

Note: Timing of assay termination should be adjusted according to the intended readout. In this study, osteoclast metabolic activity was assessed by Seahorse on day 4, 30 minutes after re-stimulation with M-CSF and RANKL. TRAP staining was performed on day 6 to evaluate osteoclast differentiation, while analysis of osteoclast activity on CaP plates was conducted on day 7 (**Figure 4***).*

### 3.5 Supplementary methods: analysis of osteoclast differentiation and function from jawbone derived precursor cells

#### 3.5.1 Quantitative analysis of multinucleated osteoclasts using TRAP staining

TRAP staining is commonly used to identify and quantify osteoclasts due to the high expression of TRAP in these cells. Although TRAP activity can also be observed in certain macrophages and dendritic cells, mature osteoclasts are distinguished by their multinucleated morphology, which arises from the fusion of mononuclear precursors. Accordingly, TRAP-positive cells containing three or more nuclei are classified as mature osteoclasts and are quantified during microscopic analysis (21). This method provides a reliable means of assessing osteoclast differentiation and maturation.

Note: All volumes are specified for a full 96-well plate and should be scaled proportionally for experiments using fewer wells or alternative plate formats. All procedures were performed at room temperature under a fume hood.

1. Prepare fixation solution.

Recipe for osteoclast fixation solution:

Mix 25 ml citrate solution with 8 ml of 36% paraformaldehyde and 65 ml acetone.

*Note: Use glass bottles for the preparation and storage of the fixation solution. The solution can be stored at 4 °C for several months. Small white crystals may precipitate during storage; avoid pipetting these crystals, as they can adhere to the wells and contribute to background staining.*

2. TRAP staining solution is prepared according to the manufacturer’s instructions (Acid Phosphatase (TRAP) Kit (Sigma-Aldrich, 387A-1KT). Add 100 µl sodium nitrite solution and 100 µl Fast Garnet solution to a 1.5ml microcentrifuge tube and mix thoroughly using a vortex mixer for 30 seconds.

3. Wrap a 15ml Falcon tube in aluminum foil to protect from light. Add 9 ml deionized water, followed by 100 µl naphthol solution, 400 µl acetate solution, and 200 µl tartrate solution.

4. Add the sodium nitrite-Fast Garnet mixture to the same tube and mix gently by inversion.

5. Osteoclast staining procedure.

*Staining is performed according to the manufacturer’s instructions.*

5.1. Remove the osteoclast culture plate from the incubator, discard the culture medium, and wash cells with 100 µl warm 1× PBS per well.

5.2. Add 100 µl fixation solution (step 1) to each well and incubate for 3 min. Wash twice with 100 µl PBS per well.

5.3. Add 100 µl staining solution (step 2) to each well and incubate at 37 °C with 5% CO_2_ for 20–30 min.

5.4. Monitor staining development and once cells appear purple, stop the reaction by washing twice with 100 µl PBS per well.

5.5. Acquire a 3 x 3 tiled brightfield image of each well at 4 x magnification and stitch the images to obtain a full-well view. Quantify the total number of osteoclasts per well using ImageJ and generate representative images of the osteoclast cultures for presentation.

#### 3.5.2 Analysis of osteoclast activity on CaP–coated plates

Calcium phosphate (CaP)-coated 96-well plates were used as an *in vitro* model of mineralized matrix to assess osteoclast-mediated lysis. Resorptive activity was quantified by visualizing lysis pits following silver nitrate staining.

1. Preparation of CaP-coated plates (adapted from (22)): Prepare CaP-coated plates by mixing 2.5 ml of 0.12 M disodium hydrogen phosphate and 2.5 ml of 0.20 M calcium chloride (prepared in 0.05 M Tris and adjusted to pH 7.4 using HCl/hydrogen chloride) under constant bubbling. Mix the solutions vigorously to prevent large aggregates. Wash the resulting CaP slurry three times with deionized water and resuspend in 38.5 ml deionized water. Dispense 100 µl of the CaP coating solution into each well of the plate, and allow the plates to dry in a closed sterile laminar floe hood for 3–4 days. Verify coating quality by phase-contrast light microscopy prior to use.

2. Seed 2 × 10^5^ cells per well (from step 3.3) onto CaP-coated plates and culture them at 37 °C in a humidified atmosphere containing 5% CO_2_ in osteoclast differentiation medium supplemented with 20 ng/ml RANKL and 30 ng/ml M-CSF.

*Note: Increased concentrations of RANKL (20 ng/ml) are necessary to achieve robust and reproducible lysis pit formation.*

3. 5 days post M-CSF and RANKL stimulation, lyse the cells by deionized water (dH_2_O), three times for 5 min each.

4. Add 100 µl of silver nitrate solution to each well and incubate for 3 h at room temperature under light exposure.

5. Wash the wells three times with deionized water (dH_2_O), leaving the water in the wells for 5 min during each wash.

6. Air-dry the plates in a chemical fume hood before imaging the lysis pits by brightfield microscopy.

7. Quantify the pit area using ImageJ software and normalize the data to the control condition to account for inter-experimental variability.

#### 3.5.3 Analysis of osteoclast metabolic function by Seahorse (Agilent Technologies) assay

Osteoclast metabolic function can be evaluated using the Agilent Seahorse XF Analyzer with the Seahorse XF Cell Mito Stress Test and Seahorse XF Glycolysis Stress Test assays. The Mitochondrial Stress Test evaluates basal respiration, ATP-linked respiration, maximal respiratory capacity, spare respiratory capacity, and non-mitochondrial respiration to determine mitochondrial function. Oligomycin inhibits ATP synthase, enabling the determination of ATP-linked respiration. FCCP uncouples the mitochondrial membrane, allowing maximal electron transport and maximal respiration to be measured. Finally, rotenone and antimycin A inhibit complexes I and III, respectively, thereby blocking mitochondrial respiration and permitting the assessment of non-mitochondrial oxygen consumption. The Glycolysis Stress Test assesses glycolytic activity, glycolytic capacity, and the metabolic response following glucose starvation.

1. Seed 1.5 × 10^5^ cells (from Step 3.3) per well into Seahorse XF 96-well cell culture microplates, preparing separate plates for the mitochondrial and glycolytic stress assays. Culture the cells in osteoclast differentiation medium supplemented with 10 ng/ml RANKL and 30 ng/ml M-CSF at 37 °C in a humidified atmosphere containing 5% CO_2_.

*Note: Use at least three technical replicates per treatment condition to ensure robust, reliable, and reproducible metabolic measurements. Additional replicates are recommended whenever possible to improve data quality and account for well-to-well variability.*

2. One day before the seahorse assay, hydrate the seahorse sensor cartridges by adding 200 µl of sterile dH_2_O to each well of the utility plate and incubate them overnight at 37 °C. Meanwhile, prepare the Seahorse assay media according to the manufacturer’s instructions **Table 1**.

**Table 1:** Medium preparation for Mito stress and glycolysis stress test.

Components	Mito stress medium	Glycolysis stress medium
Seahorse XF DMEM medium	30 ml	30 ml
Natrium Pyruvate	300 µl	–
Glutamax	300 µl	300 µl
Glucose	120 µl	–

3. 48 hours after seeding, re-stimulate the osteoclasts for 30 min at 37 °C in a humidified incubator with 5% CO_2_ using either 10 ng/ml RANKL and 30 ng/ml M-CSF alone or the same medium supplemented with the ligand under investigation. During this period, replace the dH_2_O in the hydrated Seahorse sensor cartridges with 200 µl of pre-warmed calibration solution per well and incubate the cartridges for 60 min at 37 °C in a non-CO_2_ Seahorse incubator.

4. Following stimulation, remove the culture medium and wash the cells twice with 150 µl of the appropriate Seahorse assay medium. Add fresh assay medium and equilibrate the cells for 1 h in a Seahorse incubator at 37 °C without CO_2_, leaving the plate without its lid.

5. Place the calibration plate containing the hydrated sensor cartridge into the Seahorse XF Analyzer for calibration.

6. Prepare the Seahorse assay reagents according to **Table 2** and load them into the designated ports of the hydrated sensor cartridge.

**Table 2:** Reagent concentrations for the Mito- and the Glycolysis stress test.

Mito stress test			
Reagent	Final concentration/well	Stock volume	Medium volume
Oligomycin	1 µM	300 µl	2.7 ml
FCCP	1.5 µM	450 µl	2.55 ml
Retenone/Antimycin A	0.5 µM	300 µl	2.7 ml
Glycolysis Stress Test			
Glucose	10 mM	3 ml	–
Oligomycin	1 µM	300 µl	2.7 ml
2-Deoxyglucose	5 mM	3 ml	–

*Note: The Mito Stress Test and Glycolysis Stress Test are performed as separate, independent experiments on the Seahorse Analyzer. Each assay is completed individually using a dedicated run, and the sensor cartridge is freshly prepared with the appropriate reagents before performing the subsequent assay.*

7. Load 20 µl oligomycin, 22 µl FCCP, and 25 µl rotenone/antimycin A into ports A, B, and C, respectively, of the Seahorse sensor cartridge for the Mitochondrial Stress Test.

8. Load 20 µl glucose, 22 µl oligomycin, and 25 µl 2-deoxy-D-glucose (2-DG) into ports A, B, and C, respectively, for the Glycolysis Stress Test.

9. After calibration is completed, replace the calibration plate with the Seahorse assay plate containing the cells and initiate the assay run.

10. After assay completion, determine protein content using a BCA protein assay for normalization. Remove the assay medium, wash the cells once with PBS, and add 20 µl normalization buffer per well. Normalize Seahorse readouts to a protein content of 1 µg per well.

## 4 Results

To determine whether isolated jawbone precursor cells are suitable for stable *in vitro* osteoclast differentiation comparable to established long bone protocols, we systematically assessed their differentiation, fusion capacity, resorptive function, and metabolic activity. Jawbone– and long bone derived precursors were cultured under identical M-CSF/RANKL stimulation conditions to enable direct comparison across all functional assays.

### 4.1 Comparable osteoclast precursor populations in jawbone- and long bone derived cultures after 48 h M-CSF pre-culture

During protocol optimization, a 48 h pre-culture with M-CSF (5 ng/mL), compared with the 24 h period commonly used for long-bone precursors, improved the yield and consistency of jawbone-derived osteoclast cultures. To exclude differences in pre-culture duration as a potential confounder, both jawbone- and long bone derived cells were therefore subjected to the same 48 h M-CSF pre-culture before differentiation.

To further characterize potential differences in precursor composition after the standardized pre-culture, we performed flow cytometric analysis after 48 h of M-CSF treatment. Jawbone preparations contained a lower percentage of CD45^+^CD11b^+^ myeloid cells than long bone preparations (**Figures 5A, B**). Within the CD45^+^CD11b^+^ population, however, the percentage of CD115^+^F4/80^+^ osteoclast precursors was comparable between jawbone- and long bone derived preparations (**Figures 5A, B**). These data indicate that the relative proportion of the identified osteoclast precursor population was comparable between the two preparations at the start of differentiation.

**Figure 5:**
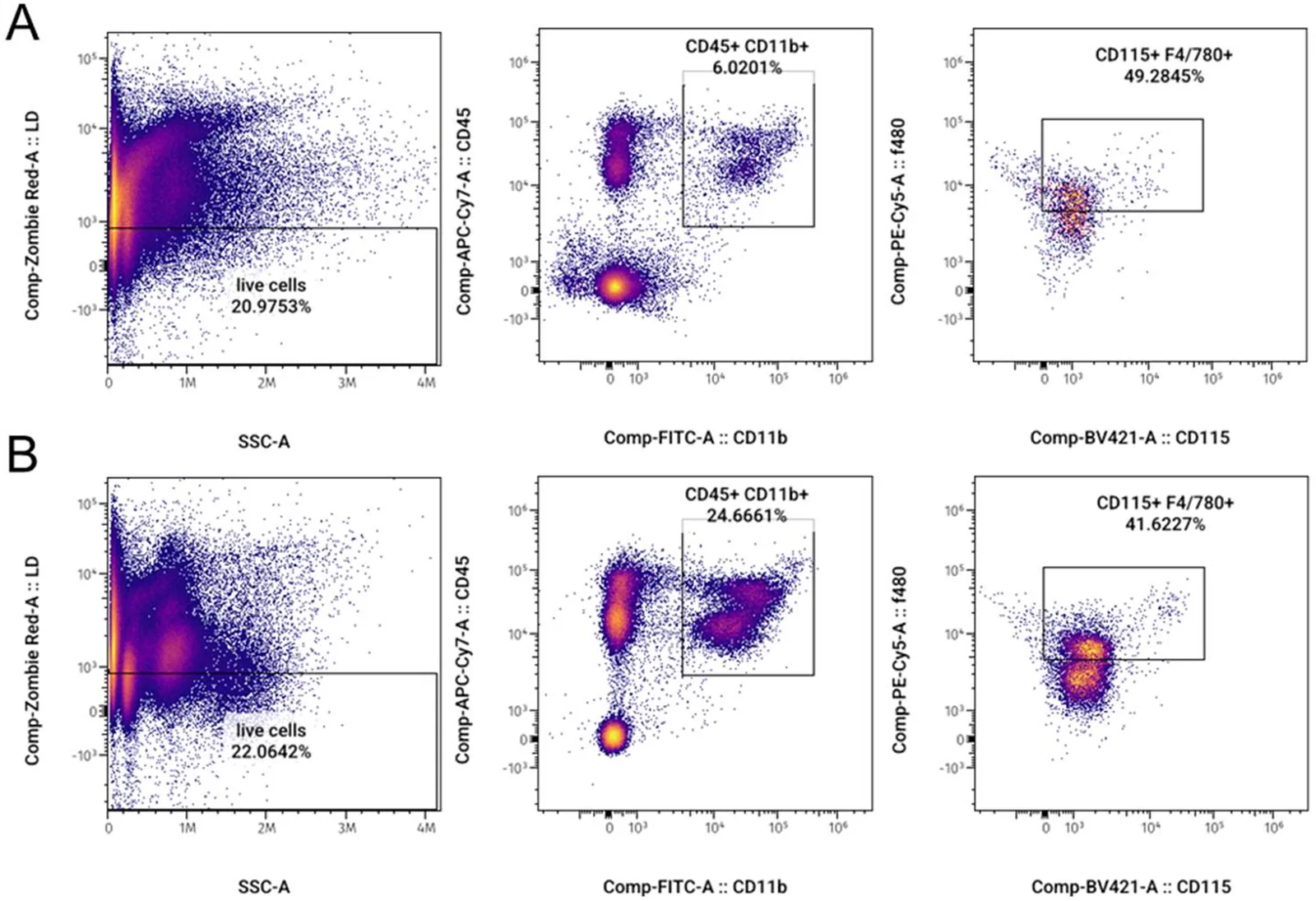
Comparable proportions of osteoclast precursors in jawbone- and long bone-derived cultures after standardized M-CSF pre-culture. Representative flow cytometry dot plots showing the percentage of CD45^+^CD11b^+^ myeloid cells and CD45^+^CD11b^+^CD115^+^F4/80^+^ osteoclast precursor cells in **(A)** jawbone-derived cultures isolated using the described enzymatic digestion protocol and **(B)** long bone-derived cells isolated using the established centrifugation-based method. Cells were analyzed after 48 h of pre-culture in osteoclast medium containing 5 ng/mL M-CSF. The experiments were performed in two independent experiments.

### 4.2 Enhanced osteoclast differentiation in jawbone-derived osteoclasts

TRAP staining performed at day 6 confirmed robust osteoclast differentiation in both groups (**Figures 6A–C**). TRAP-positive multinucleated cells were efficiently generated from jaw and long bone precursors, demonstrating that the applied protocol reliably supports osteoclastogenesis of both anatomical origins. Quantitative analysis revealed that the number of multinucleated osteoclasts was elevated in the jaw-derived cultures, indicating enhanced fusion capacity under the given conditions (**Figures 6A, B**). In addition, jawbone-derived osteoclasts displayed a more homogeneous morphology indicating robust osteoclast differentiation under the established culture conditions (**Figures 6A–C**).

**Figure 6:**
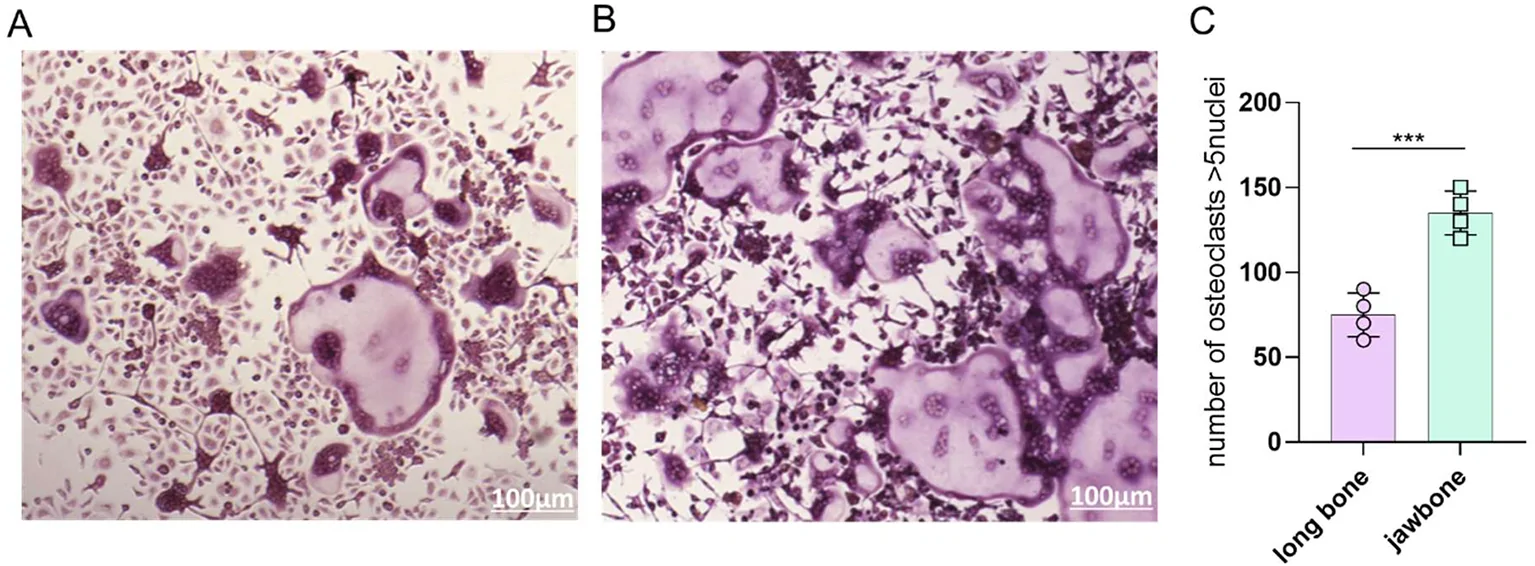
Increased formation of multinucleated osteoclasts from jawbone-derived cells. Representative images of TRAP-positive osteoclasts generated *in vitro* from **(A)** long bone marrow-derived cells and **(B)** jawbone-derived cells following 5 days of stimulation with M-CSF and RANKL. **(C)** Quantification of multinucleated TRAP-positive osteoclasts under the indicated experimental conditions. The data points are from 4 independent experiments. Data are presented as mean ± SD. Outliers were excluded using the ROUT method, and the Shapiro–Wilk test was used to test the data for normality. Statistical significance was determined using an unpaired t-test. ***P<0.001.

These results demonstrate the successful isolation and differentiation of jawbone-derived osteoclasts using the optimized protocol. Moreover, despite being generated under identical *in vitro* conditions, jawbone- and long bone-derived osteoclasts exhibited distinct morphological characteristics, including differences in cell size and multinucleation, suggesting the preservation of skeletal site-specific features. This experimental system therefore provides a robust platform for subsequent analyses of functional and metabolic differences between osteoclasts from distinct anatomical locations.

Note: Incubation time points were kept identical for bone marrow- and jawbone-derived osteoclast cultures to ensure comparability. While the workflow was based on a previously established long bone protocol (21), it was adapted and optimized for jawbone-derived osteoclast differentiation. Both systems were then analyzed under the same standardized time course to enable direct comparison.

### 4.3 Enhanced resorptive activity of jawbone-derived osteoclasts on calcium phosphate plates

To further evaluate the functional activity of jawbone-derived osteoclasts, a calcium phosphate lysis assay was performed. In this assay, osteoclast-mediated degradation of the calcium phosphate coating generates visible lysis pits, providing a measure of osteoclast activity. Osteoclasts derived from both jawbone and long bone actively lysed the mineral substrate, confirming their successful differentiation into functional osteoclasts. Notably, jawbone-derived cultures exhibited a significantly larger lysed calcium phosphate area than long bone-derived cultures (**Figures 7A–C**). This enhanced lysis likely reflects, at least in part, the greater number of osteoclasts generated in jawbone-derived cultures, consistent with the more robust osteoclastogenesis observed by TRAP staining.

**Figure 7:**
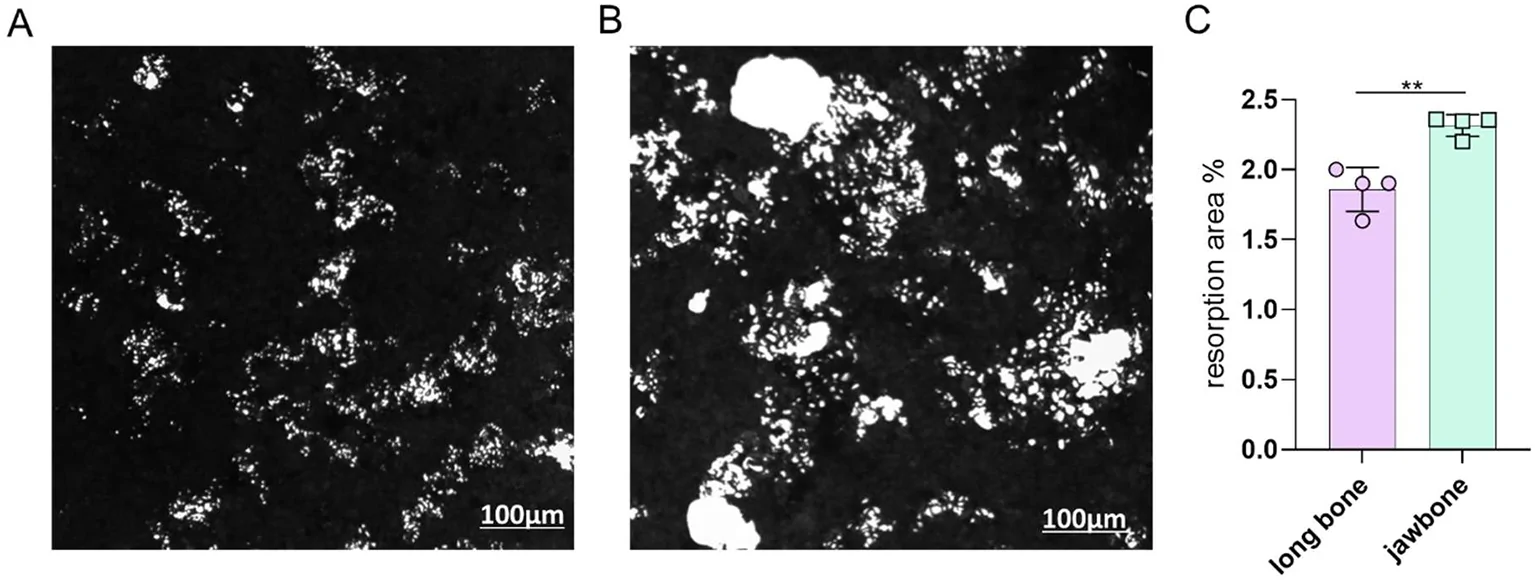
Jawbone-derived osteoclast cultures exhibit increased CaP lysis. Representative images of CaP lysis assays showing lysis pits (white) on CaP-coated plates (black) following 5 days of stimulation with M-CSF and RANKL. **(A)** Long bone marrow-derived osteoclast cultures. **(B)** Jawbone-derived osteoclast cultures. **(C)** Quantification of the total lysed CaP surface area per well under the indicated conditions. The data points are from 4 independent experiments. Data are presented as mean ± SD. Outliers were excluded using the ROUT method, and the Shapiro–Wilk test was used to test the data for normality. Statistical significance was determined using an unpaired t-test. **P<0.01.

Taken together, these findings demonstrate that osteoclasts derived from both skeletal sites are functionally active, while jawbone-derived osteoclasts exhibit enhanced osteoclastogenesis and greater activity than long bone-derived osteoclasts. The combined assessment of TRAP staining and calcium phosphate lysis provides complementary measures of osteoclast differentiation and function, validating this protocol as a robust and reproducible platform for the isolation and functional characterization of murine jawbone-derived osteoclasts.

### 4.4 Jawbone-derived osteoclasts exhibit enhanced mitochondrial respiratory capacity compared to long bone derived osteoclasts

To investigate the metabolic phenotype of osteoclast cultures, mitochondrial respiration and glycolytic activity were assessed using Seahorse XF mitochondrial (Mito) Stress and glycolytic (Glyco) stress assays. The Mito Stress Test measures the oxygen consumption rate (OCR) as an indicator of mitochondrial oxidative phosphorylation, whereas the Glyco Stress Test determines the extracellular acidification rate (ECAR) to evaluate glycolytic activity. Together, these assays provide complementary information on cellular energy metabolism. For the Mito Stress Test, OCR was recorded under basal conditions and following the sequential addition of oligomycin, FCCP, and rotenone/antimycin A. The OCR kinetics revealed distinct metabolic responses between jawbone- and long bone derived osteoclasts following 30 minutes of M-CSF/RANKL stimulation (**Figure 8A**). Jawbone derived osteoclast cultures exhibited a pronounced increase in oxygen consumption, reaching maximal OCR values of approximately 180 pmol O_2_/min after FCCP treatment, indicating enhanced mitochondrial respiratory capacity and a greater ability to meet increased energetic demands (**Figures 8A, B**). In contrast, long bone osteoclast cultures displayed only a modest increase in maximal respiration following stimulation, suggesting a more limited mitochondrial adaptive response. Quantitative analysis of mitochondrial respiration parameters further supported these findings (**Figure 8B**). Collectively, these data demonstrate that jawbone derived osteoclast cultures possess greater mitochondrial metabolic flexibility, consistent with their enhanced differentiation and lysis activity observed in the TRAP and calcium phosphate lysis assays (**Figure 8B**). Basal respiration and maximal respiration rate of jawbone derived osteoclast cultures are increased compared to long bone derived osteoclast cultures.

**Figure 8:**
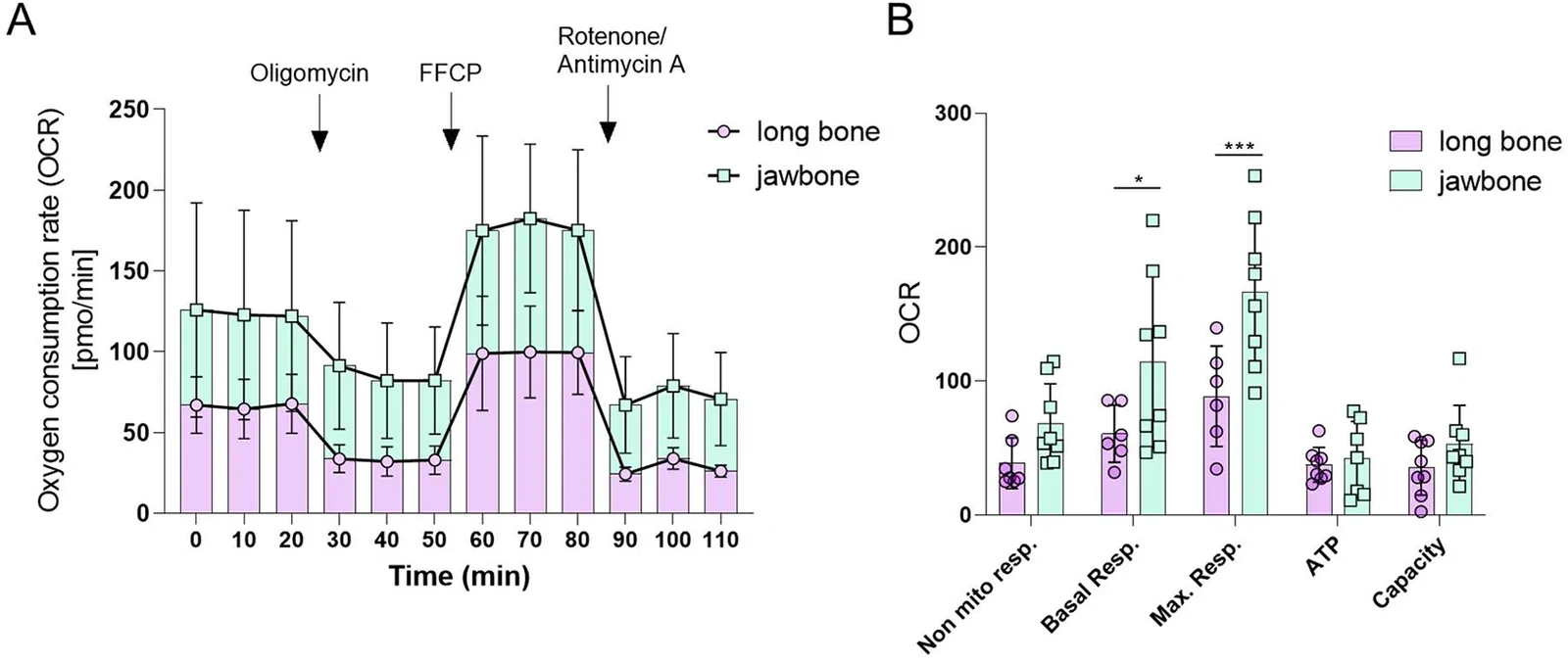
Analysis of the mitochondrial function measured as oxygen consumption rate (OCR). The Mito stress test **(A)** and the parameters of mitochondrial function **(B)** are shown for long bone derived osteoclast cultures (light purple) and jawbone derived osteoclast cultures (light green). The measured parameters are accordingly non-mitochondrial respiration, basal respiration, maximal respiration and Capacity. The data points are from 2 independent experiments. Data are the mean -/+ S.D; Two-way ANOVA was used for the analyses with Bonferroni’s multiple comparisons test. Two-way ANOVA assumptions of normality (D’Agostino-Pearson omnibus test) and homoscedasticity (Brown-Forsythe test) were met. ***P<0.005; *P<0.05.

In the Glycolysis Stress Test, cells are initially incubated in assay medium lacking glucose and pyruvate, allowing measurement of the basal extracellular acidification rate (ECAR), which primarily reflects non-glycolytic acidification. Glucose is then injected to initiate glycolysis, resulting in an increase in ECAR due to the production and export of lactate and protons. Subsequently, oligomycin, an inhibitor of mitochondrial ATP synthase, is added to block oxidative phosphorylation. As mitochondrial ATP production is inhibited, cells compensate by increasing glycolytic ATP production, leading to a further rise in ECAR. This maximal response represents the glycolytic capacity of the cells. Finally, 2-deoxy-D-glucose (2-DG), a competitive inhibitor of glycolysis, is injected to inhibit glycolytic flux, causing ECAR to decrease to non-glycolytic levels and thereby confirming that the measured acidification is glycolysis-dependent. The glycolytic reserve is calculated as the difference between glycolytic capacity and basal glycolysis and reflects the ability of cells to increase glycolytic activity in response to energetic stress.

In contrast to the marked increase in OCR observed in **Figure 8**, jawbone derived osteoclast cultures displayed no significant changes in ECAR compared with long bone derived osteoclast cultures (**Figures 9A, B**).

**Figure 9:**
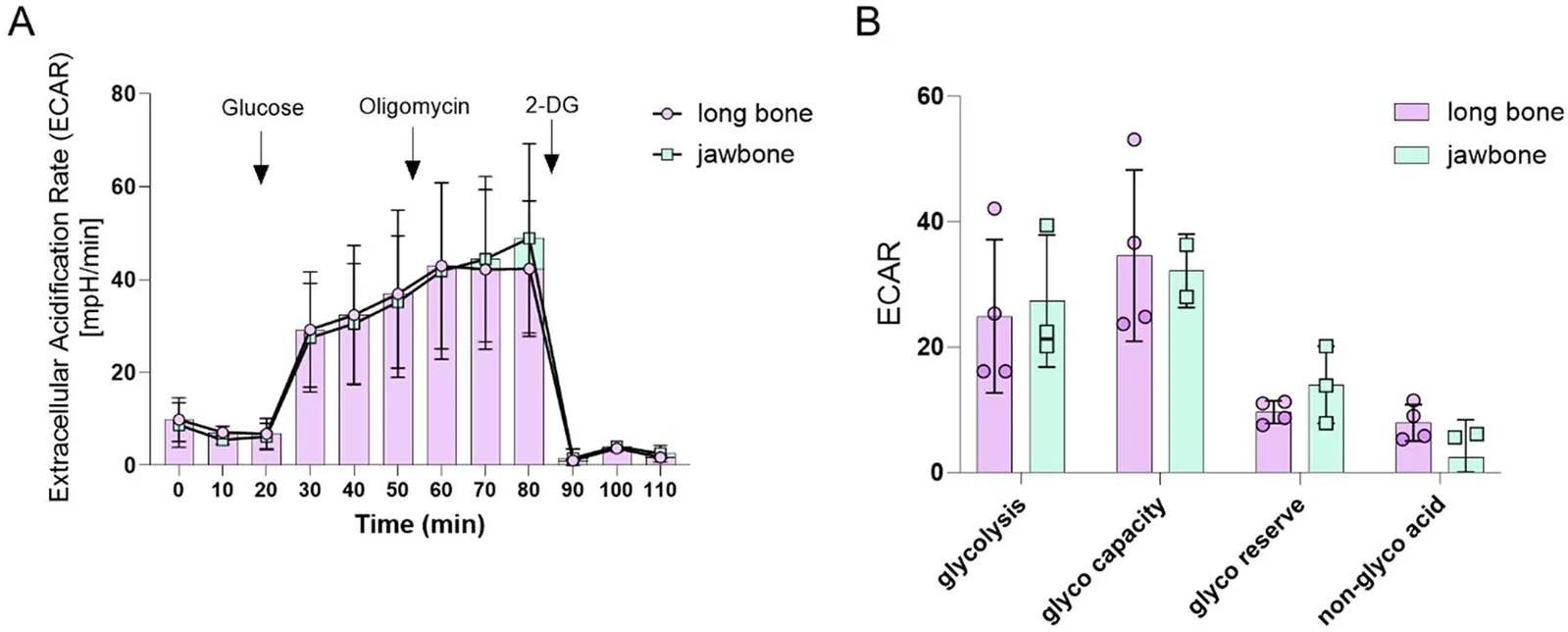
Glycolytic function of long bone- and jawbone-derived osteoclasts. Extracellular acidification rate (ECAR) was assessed using the Glycolysis Stress Test. **(A)** Representative ECAR traces and **(B)** quantification of glycolytic function in long bone derived osteoclast cultures (light purple) and jawbone derived osteoclast cultures (light green). Data represent two independent experiments. The data points are from 2 independent experiments. Data are the mean -/+ S.D;.

Collectively, these data indicate that jawbone derived osteoclast cultures preferentially rely on enhanced mitochondrial oxidative metabolism rather than increased glycolytic activity.

#### 4.4.1 Time considerations

Compared with the standard isolation of long bone marrow precursors by flushing or centrifugation, the isolation of jawbone-derived osteoclasts requires extensive mechanical disruption of the mineralized matrix, making the procedure substantially more time-consuming and labor-intensive.

#### 4.4.2 Advantages

The use of jawbone-derived osteoclasts is essential for investigating osteoclast biology in the craniofacial skeleton, as these cells more closely reflect the tissue-specific characteristics of mandibular bone remodeling than osteoclasts derived from peripheral blood or other skeletal sites. Given the well-recognized functional and developmental differences between craniofacial and appendicular bone, the use of jawbone-derived osteoclasts provides a physiologically relevant *in vitro* model for studying site-specific osteoclast responses.

Our new established protocol enables *in vitro* stimulation experiments that closely mimic the local microenvironment of the jaw, allowing detailed investigation of osteoclast differentiation, activation, metabolic adaptation, and bone-resorptive function in response to inflammatory cytokines, bacterial products, and other disease-associated signals involved in periodontal and peri-implant bone destruction. Such studies would be difficult to accurately perform using osteoclasts derived from peripheral blood or long bone, which may not recapitulate the unique biological properties of craniofacial osteoclasts.

Beyond inflammatory bone diseases, jawbone derived osteoclasts also provide a valuable platform for investigating site-specific mechanisms of pathological bone remodeling associated with conditions such as medication-related osteonecrosis of the jaw (MRONJ), osteomyelitis, and oral cancer metastasis, thereby facilitating the development of more targeted therapeutic strategies.

#### 4.4.3 Limitations, troubleshooting and possible pitfalls

This protocol describes the isolation of osteoclasts from murine jawbone; however, several limitations should be considered.

- Tissue availability and sterility.
  The success of primary cell isolation depends on tissue availability and biological variables, including the age and genetic background of the donor mice. As this protocol was established using C57BL/6 mice, cell yield and differentiation efficiency may differ in other strains. In general, the number of osteoclast precursor cells in the jawbone is limited, and incomplete recovery of bone marrow cells can further reduce the final cell yield. We therefore recommend pooling jawbone tissue from at least two mice per experiment, with four mice being preferable to ensure sufficient cell numbers for downstream applications.
  Careful tissue preparation is essential for successful cultures. All surrounding soft tissues, including gingival tissue, periosteum, muscle, and connective tissue, should be removed as completely as possible before bone processing. Residual soft tissue not only reduces the efficiency of bone marrow isolation but also increases the risk of microbial contamination, as the oral cavity harbors a high bacterial load. Importantly, this contamination risk originates from microorganisms associated with the tissue itself and therefore cannot be completely eliminated by working under sterile conditions alone. Thorough mechanical dissociation of the cleaned jawbone followed by careful filtration of the cell suspension is critical to maximize cell recovery while minimizing tissue debris and contamination.
- Culture conditions and differentiation efficiency.
  A methodological consideration is that jawbone- and long bone-derived osteoclast precursors were isolated using different established procedures, with jawbone tissue subjected to collagenase/dispase based enzymatic digestion and long bone tissue processed by centrifugation. Flow cytometric analysis after a standardized 48 h M-CSF pre-culture indicated comparable precursor populations despite the different isolation procedures. Nevertheless, the use of different isolation strategies should be considered when interpreting the site-specific comparison.
  Variability between different batches of FCS can markedly affect osteoclast survival, growth, and differentiation. FCS lot selection is therefore critical, as some batches do not adequately support osteoclast differentiation. We recommend pre-testing and selecting an FCS batch that consistently supports the complete experimental workflow to minimize batch-dependent variability. RANKL concentration should also be optimized, with a working range of 10–20 ng/ml generally providing robust osteoclast differentiation. The need for substantially higher RANKL concentrations may indicate suboptimal FCS quality. M-CSF supports the survival and proliferation of osteoclast precursors but does not induce osteoclast differentiation in the absence of RANKL. Under optimal culture conditions, multinucleated osteoclasts are typically observed after 2 days of M-CSF treatment followed by 2 additional days of combined M-CSF and RANKL stimulation, although the exact timing may vary depending on the duration and conditions of compound treatment. Cultures should therefore be monitored regularly to determine the optimal time point for TRAP staining and CaP lysis assays. If no cells are detected, the activity and quality of M-CSF should be verified. If cells are present during culture but are absent or markedly reduced after TRAP staining, the quality of the FCS batch and the fixation conditions should be evaluated.

## 5 Discussion

The present study establishes a robust and reproducible method for the isolation, differentiation, and functional characterization of jawbone-derived osteoclasts, thereby addressing a notable methodological gap in bone and periodontal research. Most *in vitro* osteoclast models still rely on long bone–derived precursors, although alveolar bone is embedded in a distinct anatomical and developmental niche and is continuously shaped by mechanical load, microbial exposure, and inflammatory challenge (13, 17). This site-specific context is highly relevant, because the cellular and molecular programs governing osteoclastogenesis in the jaw are unlikely to be fully recapitulated by conventional long bone systems (23). By enabling the generation of jawbone-derived osteoclasts under standardized conditions, our protocol provides a physiologically more relevant platform to study local osteoclast biology and to dissect alveolar bone remodeling in health and disease.

A major strength of this method is that it enables controlled, reductionist analysis of osteoclast differentiation and function while preserving site-specific characteristics during the limited *in vitro* culture period, features that are otherwise difficult to dissect in complex *in vivo* models (19). This is particularly important in periodontitis, where dysbiosis, persistent inflammation, and excessive osteoclast activity converge to drive alveolar bone loss (1, 2, 24). Oral microorganisms and their ligands, including β-glucans and mannans, engage host PRRs and shape the inflammatory milieu, while osteoclast lineage cells themselves can sense microbial and danger-associated signals through PRRs, thereby integrating innate immune cues into osteoclastogenic signaling networks (3, 10, 11). In this context, jawbone-derived osteoclasts are especially valuable because they may better reflect the functional adaptation of the alveolar compartment to its chronically exposed microenvironment. Our findings are consistent with the concept of site-specific osteoclast heterogeneity and extend previous evidence that osteoclast precursors can differ across skeletal niches (13, 25). Importantly, such differences may reflect both intrinsic properties of site-specific precursor populations and their selective recovery or adaptation during *ex vivo* culture. Beyond differentiation efficiency, the main value of the system lies in its reproducibility and experimental control, which allow direct comparison with long bone-derived osteoclasts and mechanistic interrogation of conserved and site-adapted pathways, including RANKL-NFATc1 signaling, as well as the effects of inflammatory, microbial, and metabolic cues on resorptive function in a jaw-specific context (8).

Importantly, our findings also need to be considered in the context of previous studies reporting differences in the morphology and resorptive activity of long bone- and jawbone-derived osteoclasts. In one such study, long bone-derived osteoclasts were reported to be larger than jawbone-derived osteoclasts when differentiated on plastic; however, when osteoclast function was assessed on dentin, jawbone-derived osteoclasts exhibited greater cell size while showing comparable resorptive activity to their long bone-derived counterparts (19). In contrast, in our experimental system, jawbone-derived osteoclasts were consistently larger on plastic and displayed enhanced lysis activity on calcium phosphate substrates compared with long bone-derived osteoclasts. These apparently divergent findings may reflect differences in the intrinsic properties of osteoclast precursors, but could also be influenced by the isolation procedures, culture conditions, and substrates used for functional assessment (26, 27). In particular, the composition and state of the precursor population recovered during tissue dissociation may affect the phenotype of the resulting osteoclasts. In the present study, jawbone and long bone precursors were isolated using different established procedures, namely collagenase/dispase digestion for jawbone and centrifugation for long bone. Preliminary attempts to apply enzymatic digestion to long bone resulted in increased apoptosis and reduced cell recovery and did not yield sufficiently robust and reproducible cultures for inclusion in the present study. We therefore retained the established site-specific isolation procedures. Importantly, flow cytometric analysis following a standardized 48 h M-CSF pre-culture revealed comparable precursor populations, providing support that the observed differences in osteoclast differentiation are not simply attributable to major differences in the recovered precursor populations. Nevertheless, comparable flow cytometric profiles do not exclude more subtle differences in precursor maturation state, activation history, viability, tissue retention, or transcriptional and epigenetic programming (28). Previous studies have reported skeletal-site-specific differences in myeloid and osteoclast precursor populations, including populations defined by CD11b and related markers (13, 29). Thus, while our data support the comparability of the isolated populations, they cannot establish complete biological equivalence or fully exclude effects of the different isolation procedures. The enhanced osteoclast size and lysis activity observed in jawbone-derived cultures may therefore reflect a combination of anatomical site-specific properties and methodological effects.

The relationship of our optimized protocol to previously published methods also warrant consideration. Our approach was adapted from a previously described enzymatic digestion-based method (20) and further optimized with respect to tissue preparation, enzymatic digestion, cell seeding, and M-CSF/RANKL conditions. In addition, the present protocol provides detailed procedures for reproducible osteoclast differentiation as well as functional and metabolic analyses. The main contribution of the present study is therefore to provide a detailed and optimized workflow that yielded reproducible jawbone-derived osteoclast cultures and enabled their morphological, functional, and metabolic characterization in our experimental setting.

At the same time, the present method should be interpreted with appropriate caution. As with any *in vitro* system, it cannot fully reproduce the spatial complexity of periodontal tissues, including epithelial barriers, vascular and neural inputs, biomechanical forces, and the dynamic host–microbe interactions that occur *in vivo* (30, 31). Accordingly, the protocol is not intended to replace ligature- or bacteria-induced periodontitis models, which remain essential for defining tissue-level disease mechanisms and therapeutic responses. Rather, its added value lies in complementing these models by enabling mechanistic dissection of cell-intrinsic osteoclast biology under defined conditions. In that sense, the method fills an important gap between simplified marrow-derived culture systems and the biological complexity of intact periodontal disease models.

In conclusion, this study provides more than a technical refinement: it establishes a physiologically relevant and reproducible *in vitro* system for investigating jawbone-derived osteoclasts. By enabling experimental access to a skeletal niche that is continuously exposed to inflammatory and microbial stress, the method offers new opportunities to study the cellular basis of alveolar bone loss and to identify mechanisms that may be therapeutically targetable in periodontitis and related disorders.

## Data Availability

The original contributions presented in the study are included in the article/**Supplementary Material**. Further inquiries can be directed to the corresponding author.
